# Integration of multiomics analysis to reveal the major pathways of vitamin A deficiency aggravates acute respiratory distress syndrome in neonatal rats

**DOI:** 10.1038/s41598-023-47664-x

**Published:** 2023-12-19

**Authors:** Jia Tang, Jiaqin Yuan, Jinghao Sun, Mi Yan, Mengchun Li, Yanfei Liu, Shaohua Xu, Jing Li, Hong Fu, Wanwei Li, Zhangxue Hu

**Affiliations:** 1 Department of Pediatrics, Daping Hospital, Army Medical University, Chongqing, 400042 China; 2 Department of Pediatrics, Chongqing University Jiangjin Hospital, Chongqing, 402260 China; 3 Department of Orthopedics, The Second People’s Hospital of Yibin, Yibin, 644000 China; 4 Department of Clinical Laboratory, Center for Gene Diagnosis and Program of Clinical Laboratory, Zhongnan Hospital of Wuhan University, Wuhan, 430071 China

**Keywords:** Health care, Medical research

## Abstract

Acute respiratory distress syndrome (ARDS) is a major disease that threatens the life and health of neonates. Vitamin A (VA) can participate in early fetal lung development and affect lung immune function. Researches revealed that the serum VA level in premature infants with ARDS was lower than that in premature infants without ARDS of the same gestational age, and premature infants with VA deficiency (VAD) were more likely to develop ARDS. Moreover, the VA levels can be used as a predictor of the development and severity of neonatal ARDS. However, the critical question here is; Does ARDS develop due to VAD in these systemic diseases? Or does ARDS develop because these diseases cause VAD? We hypothesize that VAD may aggravate neonatal ARDS by affecting immunity, metabolism, barriers and other pathways. In this article, we used multiomics analysis to find that VAD may aggravate ARDS mainly through the Fc epsilon RI signaling pathway, the HIF-1 signaling pathway, glutathione metabolism, and valine, leucine and isoleucine degradation signaling pathways, which may provide the molecular pathogenic mechanism behind the pathology of VAD-aggravated ARDS and can also provide potential molecular targets for subsequent research on ARDS.

## Introduction

Acute respiratory distress syndrome (ARDS) is one of the major diseases that threatens the life and health of neonates^1^. Among children hospitalized in the neonatal intensive care unit (NICU), the morbidity and mortality of neonatal ARDS is high^2^. Its clinical manifestations are shortness of breath, respiratory distress, hypoxemia, decreased light transmittance in both lungs and even white lung^3^. As an essential micronutrient for human metabolism, vitamin A (VA) can participate in early fetal lung development and affect lung immune function^4^. VA deficiency (VAD) has a high incidence worldwide, with up to 140 million preschool children exhibiting VAD^5^. A previous study revealed that the serum VA level in premature infants with ARDS was lower than that in premature infants without ARDS of the same gestational age, and premature infants with VAD were more likely to develop ARDS^6^. Elfarargy et al found that VA levels can be used as a predictor of the development and severity of neonatal ARDS^7^. It is suggested that VAD is closely related to the severity of neonatal ARDS. However, the critical question here is; Does ARDS develop due to VAD in these systemic diseases? Or does ARDS develop because these diseases cause VAD? and the existing research results have not yet elucidated the mechanism by which VAD aggravates ARDS. We hypothesize that VAD may aggravate neonatal ARDS by affecting immunity, metabolism, barrier and other pathway. Besides, not all complex features of ARDS and coexisting diseases can be demonstrated in animal models. Despite these challenges, it is still need to uncover numerous genomic, metabolomic, and proteomic pathways to understand the pathophysiology of ARDS, which will provide a better understanding of the molecular pathogenetic disorders underlying pathologies of ARDS.

In this study, neonatal VAD rats were used to establish an ARDS neonatal rat model, and then we explored the role and mechanism of VAD in regulating ARDS through multiomics analysis. Elucidating the regulatory effect and mechanism of VAD on neonatal ARDS can offer a reference for the prevention and treatments of the disease, which is of great importance for improving the prognosis of neonatal ARDS.

## Materials and methods

This experiment was reviewed by the Animal Ethics Committee of the Army Specialty Medical Center and completed based on the requirements of the Animal Ethics Committee (AMUWEC20219010). All experiments were performed in accordance with relevant guidelines and regulations. Besides, the study is reported based on the ARRIVE guidelines.

### Preparation animals models

VAD and VA normal (VAN) neonatal rat model preparation: Two weeks before pregnancy, female rats were fed the VAN diet (2300 IU VA/kg, n = 20) and VAD diet (400 IU VA/kg, n = 20); male rats(n = 20) were fed a common conventional diet^8^. The two groups of female rats were mated in cages according to the ratio of female:male = 2:1 to obtain VAD neonatal rats and VAN neonatal rats^9^.

The experiments were divided into 4 groups (per group n = 20): VA normal control group (VAN-C), VA deficiency control group (VAD-C), VA normal experimental group (VAN-E), VA deficiency experimental group (VAD-E). Neonatal rats in VAN-C and VAD-C group were given Phosphate Buffered Saline (PBS) in the trachea, while the pups in VAN-E and VAD-E group were given Lipopolysaccharide (LPS, Sigma, USA, serotype 055:B5, 5 mg/kg) in the trachea to establish a neonatal rat ARDS model^10^.

Neonatal 7 day SD rats were performed with LPS or PBS. The skin was open in the middle of the neck to expose the trachea. LPS solution was injected into the rat airways, and the cut was sutured. While for the VAN-C and VAD-C group, trachea was exposed and PBS was performed, then the cut was sutured. After 4 hours, the neonatal rats were placed supine on the operating table and anesthetized with 1% pentobarbital sodium 5 mL/kg intraperitoneally. The right upper lobe of the rat lung was taken and the left lung of each sample were divided into 3 parts respectively and labeled for transcriptomics, proteomics and metabolomics detection. Samples were collected and placed on dry ice.

### General condition and pathological observation of neonatal rats

The general condition and lung injury of neonatal rats in each group were recorded after LPS conducted for 4h. After modeling, lung tissue samples were taken to observe the hemorrhage and edema (HE) of the lung tissue in the neonatal rats in each group^11^. Lung wet-dry (W/D) ratios were detected. The right upper lobe of the rat lung was taken, and the weight of the specimen was measured. The samples were then placed in a 60 °C oven and dried for 72 h. The W/D ratio was calculated by comparing the weight of the lung lobes immediately after removal with the weight after drying and was expressed as wet W/D weight.

### Measurement of the levels of VA in serum, blood gas in lung tissue

Blood collected through the severed head from the rats in each group were separated at 3000 rpm for 10 min, and the serum (supernatant) was taken and diluted with PBS, and the VA content in the rats in each group was measured^12^. Serum VA content was measured using Elisa kit. the blood samples of rats collected were performed at 3000 rpm, separated for 10 min, and the upper serum was taken according to the instructions of the Elisa kit (Jiangsu Jingmei Biotechnology, Jiangsu, China.), then the standard curve was drawn after the OD value was measured to calculate the concentration value of each sample. For the blood gas analysis, 1 mL heparin sodium was administered to flush the needle and tubes, and then about 0.3–0.6 mL of blood was collected for detection from the other rats through the left heart^13^.

### Transcriptomics analysis

RNA sequencing (RNA-Seq) and gene expression analysis were performed by BioNovoGene (Suzhou, China). Total RNA was isolated using the Trizol Reagent (Invitrogen Life Technologies), after which the concentration, quality and integrity were determined using a NanoDrop spectrophotometer (Thermo Scientific). Then, First-strand cDNA was synthesized using RNA as a template, and second-strand cDNA was synthesized using first-strand cDNA as a template. To select cDNA fragments of the preferred 400–500 bp in length, the library fragments were purified using the AMPure XP system (Beckman Coulter, Beverly, CA, USA). The reference genome and gene annotation files were downloaded from genome website. The filtered reads were mapping to the reference genome using HISAT2 v2.0.5.Then, the quality of the library was evaluated by an Agilent 2100 Bioanalyzer (Agilent Technologies, Santa Clara, CA, United States).After RNA extraction, purification, and library construction of the samples, next-generation sequencing technology was used to perform paired-end (PE) testing on these libraries using the Illumina sequencing platform (NEB, United States)^14^. Then difference expression of genes was analyzed by DESeq (1.30.0) with screened conditions as follows: expression difference multiple |log2FoldChange| > 1, significant P-value < 0.05. At the same time, We used R language Pheatmap(1.0.8) software package to perform bi-directional clustering analysis of all different genes of samples^14^. On the basis of the existing reference genome, using the software StringTie (http://ccb.jhu.edu/software/stringtie/) to assemble the mapped reads. Next, using topGO to perform GO enrichment analysis on the differential genes, calculate P-value by hypergeometric distribution method, and find the GO term with significantly enriched differential genes to determine the main biological functions performed by differential genes. ClusterProfiler (3.4.4) software was used to carry out the enrichment analysis of the KEGG pathway of differential genes. All original sequence datasets have been submitted in the supplementary tables.

### Proteomics analysis

The rat lung samples were homogenized using SDS protein lysis buffer by vortex oscillation and passaged using a high-throughput tissue grinding machine thrice. The sample was collected after centrifugation at 12000g at 4 °C for 20 min. The concentrations of the supernatant were measured with a BCA Protein Assay Kit. An appropriate amount of protein was added to a final concentration of 5 mM DTT and incubated at 37 °C for 1 h. Iodoacetamide was added at a concentration of 10 mM and incubated at 25 °C for 45 min in the dark. The supernatant was diluted 4 times with 25 mM ammonium bicarbonate, trypsin was added at a 50:1 ratio of protein to trypsin, and the mixture was incubated overnight at 37 °C. The next day, formic acid was added to adjust the pH to less than 3 to terminate the digestion. Samples were desalted using a C18 desalting column. The TMT reagent was removed and thawed at 25 °C, the lid was opened, and 41 μl of acetonitrile was added followed by centrifugation. The TMT reagent was added to 100 μg of the digested samples, and the reaction was carried out at 25 °C for 1 h. Ammonia was used to terminate the reaction. The specimens were mixed, vortexed, and centrifuged. Vacuum freeze centrifugation drying was performed. The mixed and labeled samples were dissolved in 100 μl of mobile phase A and centrifuged at 14,000×g for 20 min. Then, the sample was collected and processed by a high-performance liquid phase. Then, liquid chromatography with tandem mass spectrometry (LC–MS/MS) analysis was conducted by online nanospray LC–MS/MS on an Orbitrap Exploris™ 480 mass spectrometer (Thermo Fisher Scientific, MA, United States) coupled to an EASY-nanoLC 1200 system (Thermo Fisher Scientific, MA, United States). Spectronaut 13 (Biognosys AG, Switzerland) was used to process and analyze the raw DIA data. Proteins were analyzed using gene ontology (GO), Kyoto Encyclopedia of Genes and Genomes (KEGG) and Clusters of Orthologous Groups/ EuKaryotic Orthologous Groups (COG/KOG) analyses to determine their functions. Proteins with a Q value < 0.05 and absolute AVG log2 ratio > 0.58 were considered differentially expressed proteins^15^.

### Metabolomics analysis

The lung samples were pretreated for LC‒MS analysis. The lung samples were subjected to quality control (QC) and LC‒MS detection. The raw data were first converted to mzXML format by MSConvert in the ProteoWizard software package (v3.0.8789) and processed using XCMS for feature detection, retention time correction and alignment. Base peak chromatograms (BPCs) were obtained through a continuous description of the ions with the highest intensity in each mass spectrogram. Data were analyzed using QC and quality assurance (QA). After scaling the data, models were established using principal component analysis (PCA), orthogonal partial least square discriminant analysis (OPLS-DA) and partial least- square discriminant analysis (PLS-DA). The metabolic profiles were visualized as a score plot in which each point represents a sample. The corresponding loading plot and S-plot were generated to provide information on the metabolites that influenced the clustering of the samples. All the models evaluated were tested for overfitting with permutation tests. Differential metabolites were subjected to pathway analysis by MetaboAnalyst, which combines the results from powerful pathway enrichment analysis with the pathway topology analysis. The identified metabolites in metabolomics were then mapped to the KEGG pathway for biological interpretation of higher-level systemic functions. The metabolites and corresponding pathways were visualized using the KEGG Mapper tool^16^.

### Integrated multiomics analysis

In short, the genes, proteins, and metabolites were evaluated. However, further multiomics integrated analysis was needed for in-depth functional study and validation of potential biomarkers^17^. Moreover, the biological effect of VAD on ARDS on the lung needs to be explored at the pathway level rather than focus excessively on individual molecules, which would prevent the elucidation of the causative context. We conducted integrated multiomics analysis of KEGG pathways to investigate the mechanism of VAD in ARDS from a holistic perspective. Overall, the integrated multiomics analysis identified multiple genes and pathways that potentially clarify the role of VAD in ARDS.

### Statistical analysis

**Figure 1:**
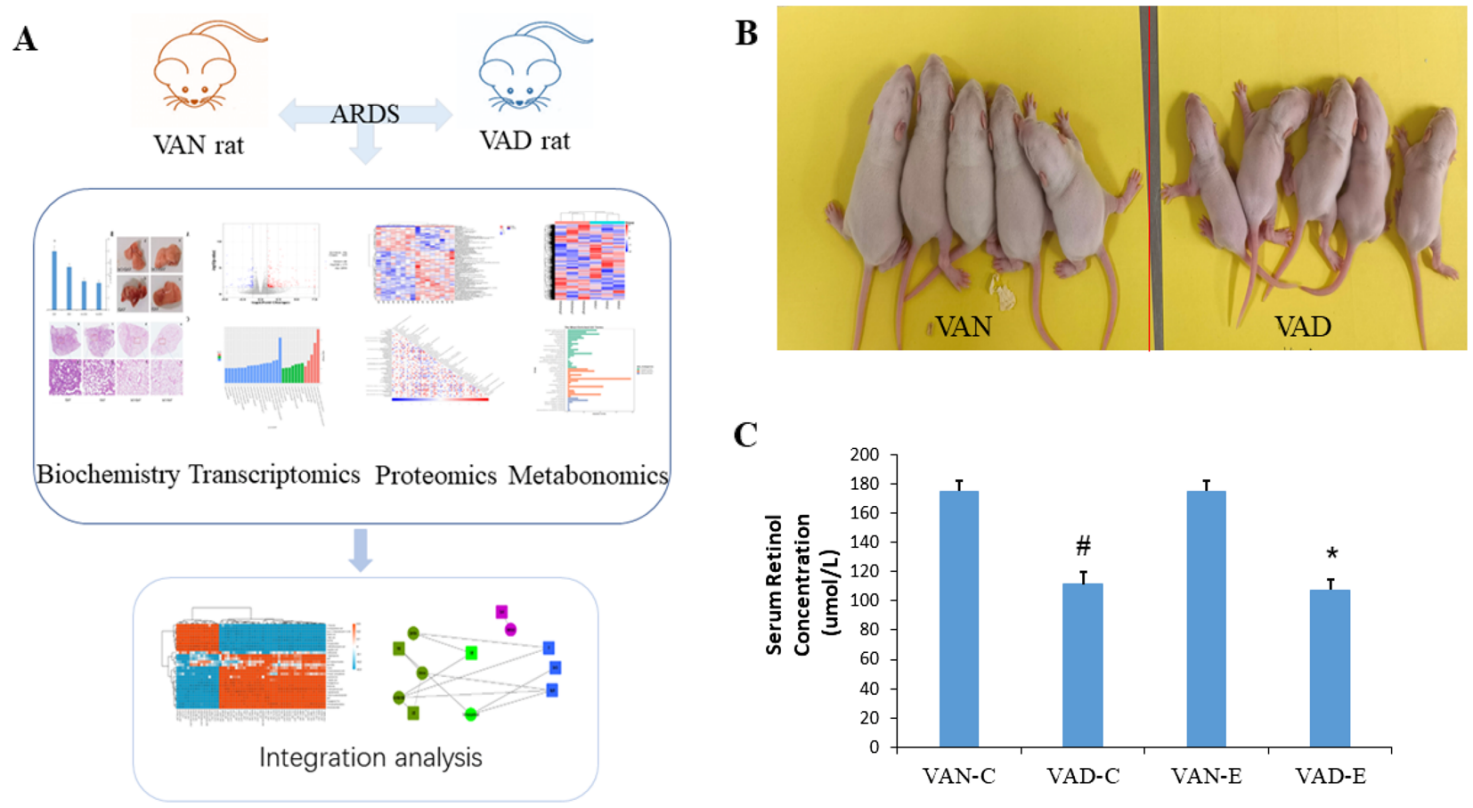
Experimental process and VAD model establishment. (**A**) Experimental flow chart. (**B**) Morphology of VAN and VAD mice. (**C**) Serum VA content n = 8. *means, the VAD-E group vs the VAN-E group, P < 0.05. # means, the VAD-C group vs. the VAN-C group, P < 0.05.

The results are shown as the means ± standard deviation (SD). Statistical analysis was performed by SPSS 22. One-way ANOVA with least significant difference (LSD) and unpaired Student’s t-test were performed for analysis. Results with a p value < 0.05 were regarded as statistically significant. VAD neonatal rat models building. The experimental procedure is shown in Fig. 1A. Neonatal 7-d rats were used for modeling. Before modeling, VAD neonatal rats were smaller, lighter and had more sparse hair than VAN neonatal rats (Fig. 1B), and VAN neonatal rats were generally in a better condition, with smooth hair and well developed (Fig. 1B). The serum VA content in the four groups in rats was measured, and it was found that the serum VA content in the VAD-C group was significantly lower than that in the VAN-C group (Fig. 1C) (P < 0.05), and the serum VA content in the VAD-E group was significantly lower than that in the VAN-E group (P < 0.05) (Fig 1C).

### Neonatal ARDS rats models building

**Figure 2:**
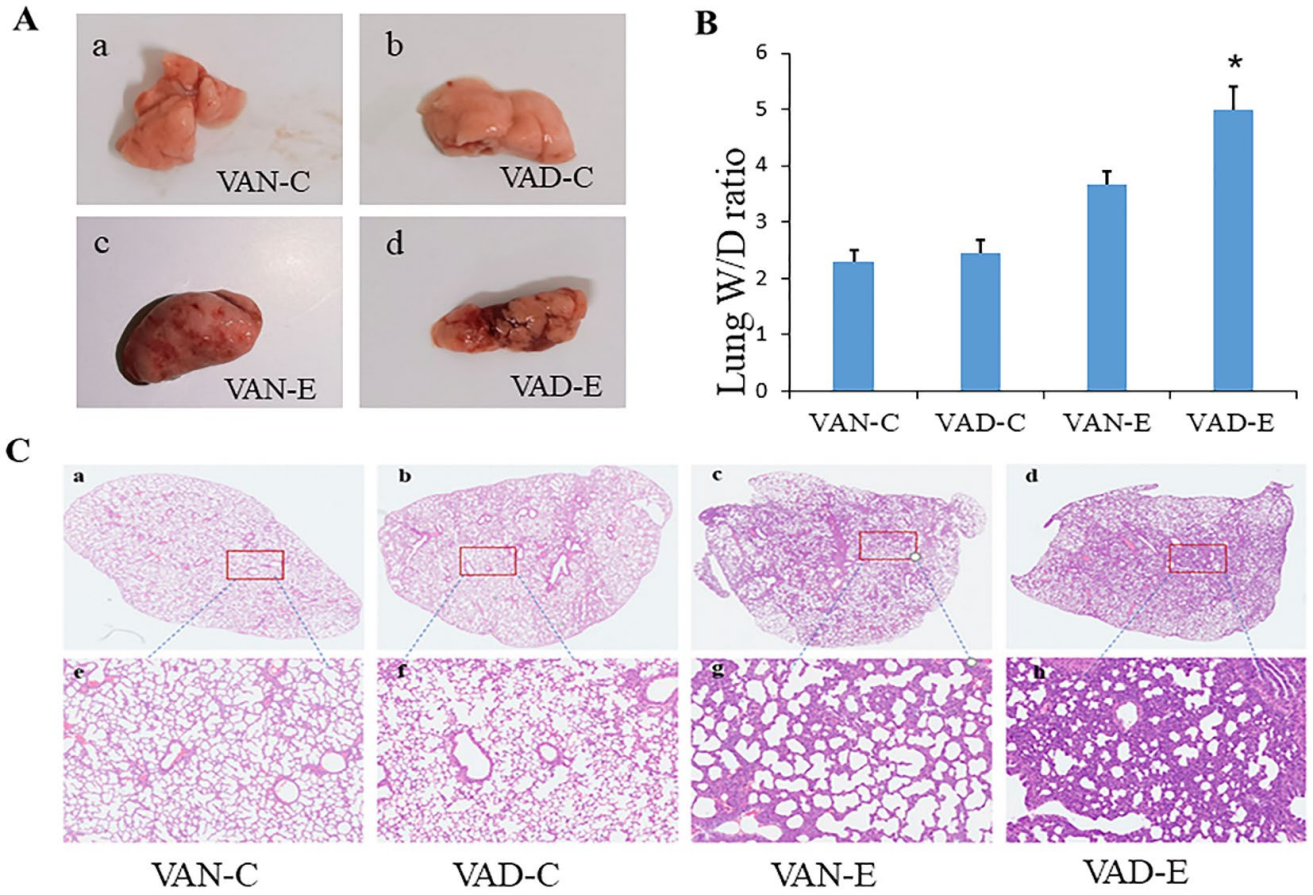
VAD aggravates lung injury in ARDS. (**A**) General morphology of lung injury in mice from each group. (**B**) W/D ratio in lung injury. (**C**). Histological scores n = 8. *means, the VAD-E group vs the VAN-E group P < 0.05. (a–d 2×, e–h 40×), # means VAD-C group vs the VAN-C group, P < 0.05.

**Figure 3:**
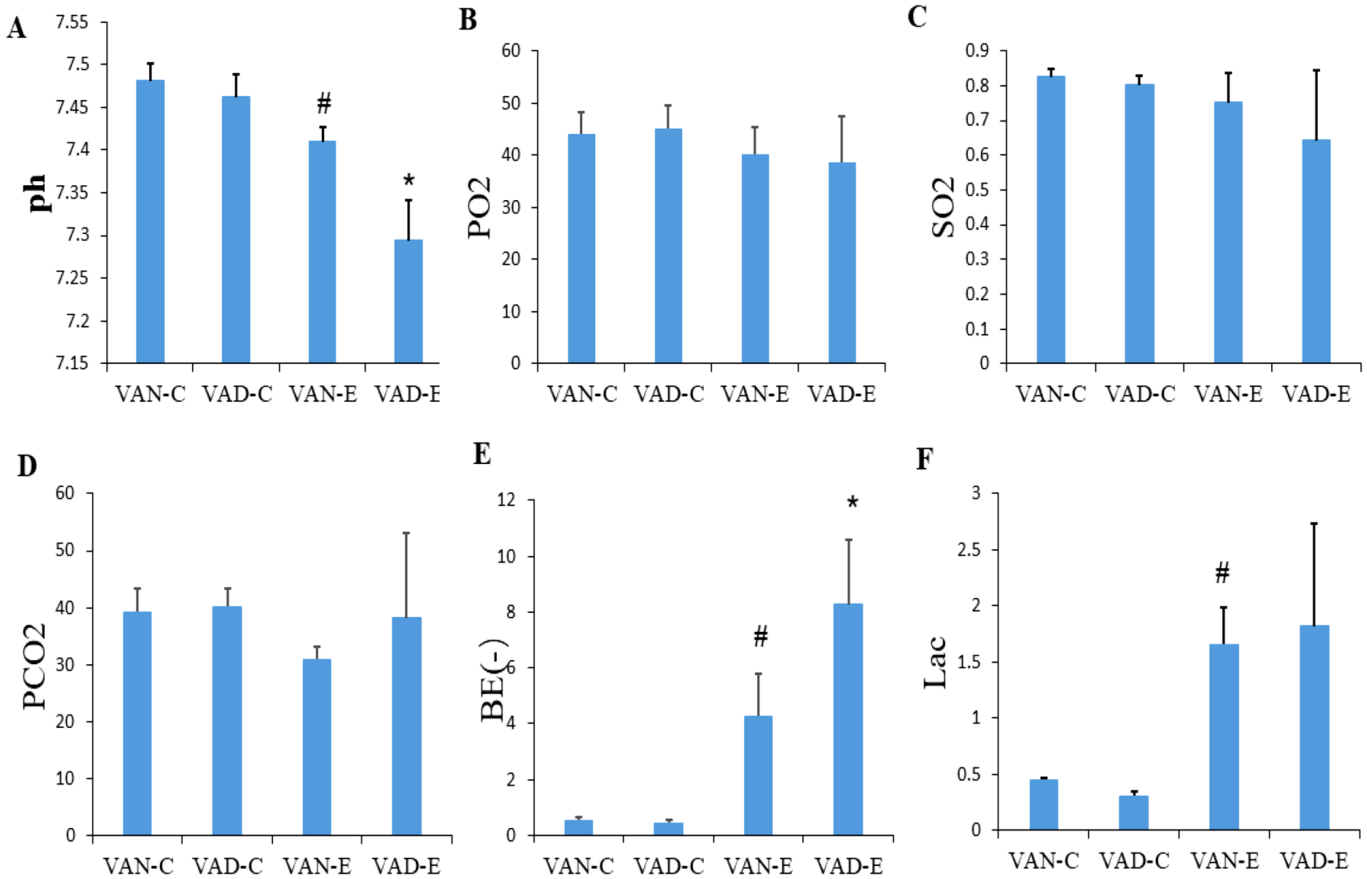
Blood gas analysis in mice from each group. (**A**) pH value. (**B**) Oxygen partial pressure. (**C**) Blood oxygen saturation. (**D**) Partial pressure of carbon dioxide. (**E**) BE value. (**F**) Lactic acid value, n = 8. *means, the VAD-E group vs the VAN-E group, P < 0.05. # means VAD-C group vs the VAN-C group, P < 0.05.

Neonatal rats in both the VAD-E and VAN-E groups showed symptoms such as shortness of breath, respiratory distress, and cyanosis after LPS modeling. Upon specimen examination, both the two groups showed lung tissue congestion and edema, suggesting neonatal ARDS (Fig. 2A–C). However, the injury of the rats in the VAD-E group, which showed a higher W/D ratio, was worse than that in the VAN-E group. Our results showed that the blood gas parameters in the VAD-E group were worse than those in the VAN-E group, which manifested as a lower pH value (Fig. 3A) and a lower blood oxygen saturation level (Fig. 3B,C), while the partial pressure of carbon dioxide (Fig. 3D), the BE value (Fig. 3E) and lactic acid levels were higher (Fig. 3F) in the VAD-E group. The BE value exhibited the most significant difference between the VAD-E and VAN-E groups. However, there were no statistical differences in the HE staining and blood gas results between the VAN-C and VAD-C group.

### Transcriptome analysis

**Figure 4:**
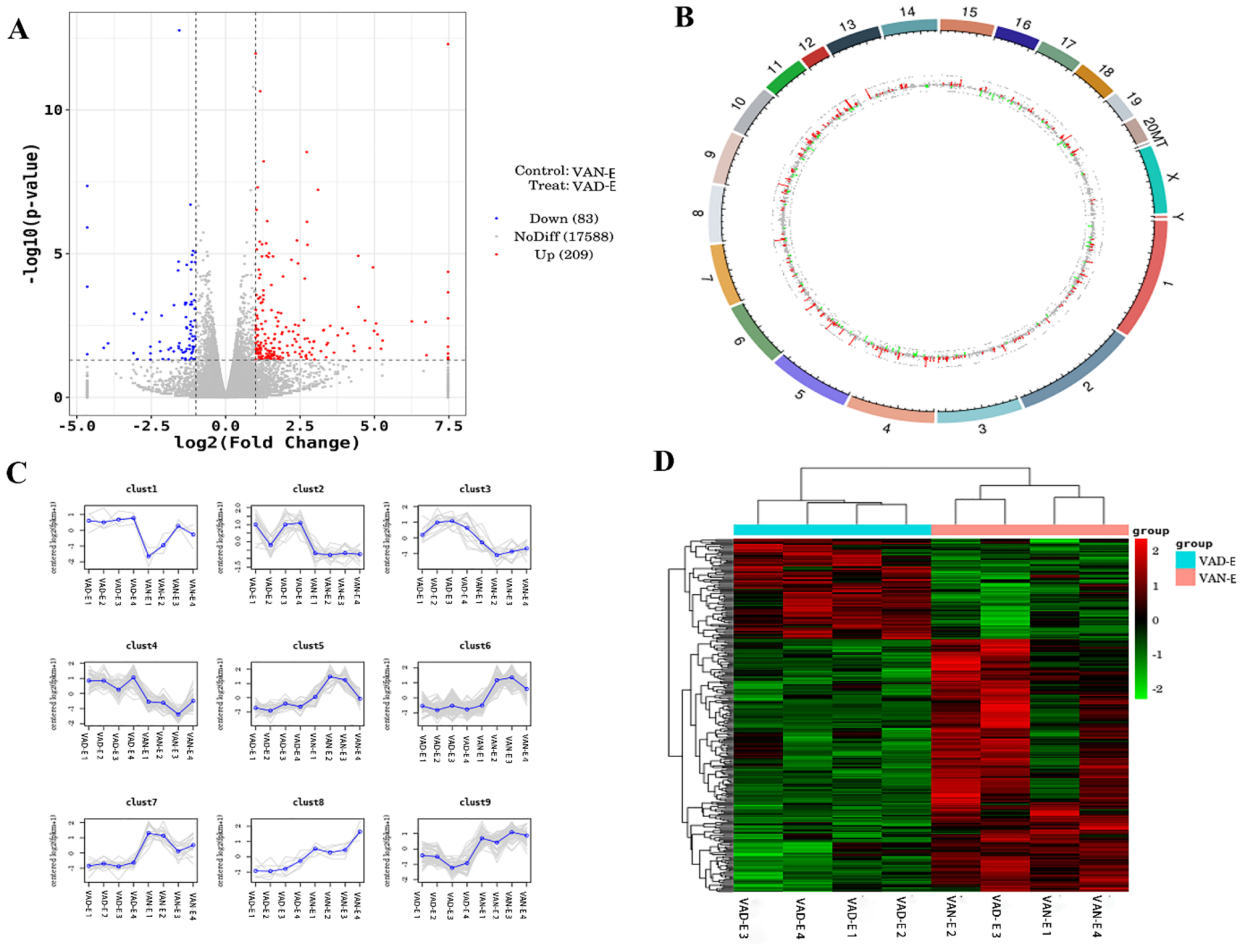
Analysis of the transcriptomic data. (**A**) Volcano plots showing relative transcript abundances. (**B**) Genome circular map. The outermost circle is the chromosome band; the differential expression analysis results of different differential analyses are shown from outside to inside. (**C**) Trend analysis. (**D**) Clustering of differentially expressed genes. n = 4.

**Figure 5:**
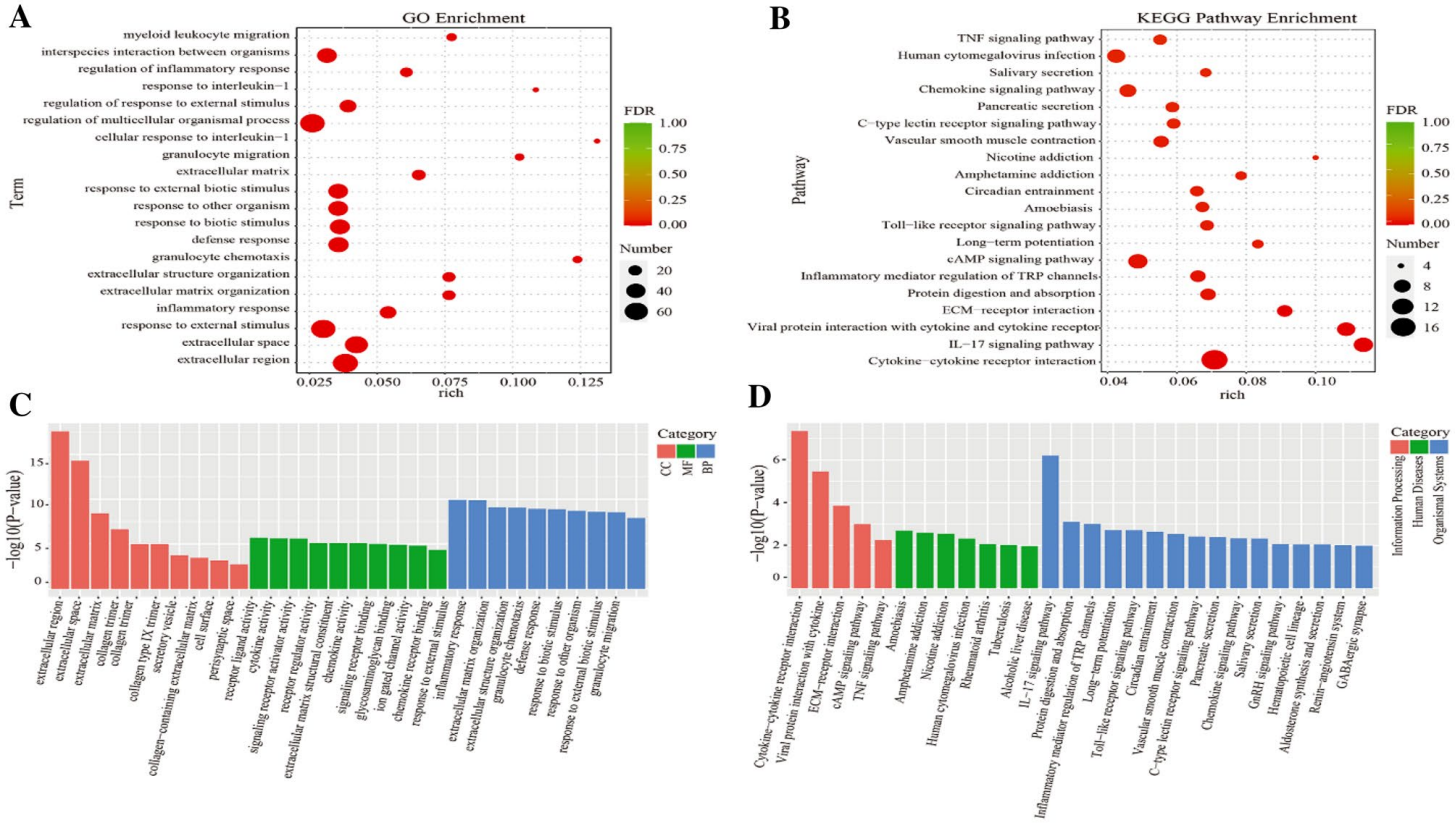
Analysis of the transcriptomic data. (**A**) KEGG pathway analysis of pathway enrichment. (**B**) Histogram of KEGG pathway enrichment results. (**C**) GO pathway analysis of pathway enrichment. (**D**) GO enrichment analysis histogram.

RNA-seq analysis was performed to investigate the gene expression changes^18^. Raw Data are uploaded on SRA database and the processed data are showed in the supplementary table 1. (https://submit.ncbi.nlm.nih.gov/subs/sra/SUB13512004, PRJNA982530).The differentially expressed genes (DEGs) between the VAD-E and VAN-E animals are shown in volcano plots (Fig. 4A). The results showed that the gene expression profiles differs dramatically between VAN-E and VAD-E groups (Fig. 4B–D). We chose the genes that were differentially expressed with fold changes (FC) > 2 and with statistically significant differences between the two groups. Overall, 292 genes were demonstrated to be differentially expressed, among which 83 genes exhibited decreased levels and 209 genes exhibited increased levels. Then, we further explored the potential biological functions of the DEGs. The KEGG pathway enrichment analysis results showed that the differentially expressed genes mainly included genes involved in cytokine‒cytokine receptor interactions, the Fc epsilon RI signaling pathway, vitamin digestion and absorption, the HIF-1 signaling pathway, viral protein interactions with cytokines and cytokine receptors, ECM-receptor interactions, protein digestion and absorption, and cAMP signaling pathways (Fig.5A-B). GO analysis showed that the DEGs clustered in biological processes involved in the regulation of multicellular organismal processes, responses to external stimuli, interspecies interactions between organisms, and extracellular regions (Fig. 5C-D). Our results showed that different pathways were triggered by VAD experiment in the ARDS rat model.

### Proteomics analysis

**Figure 6:**
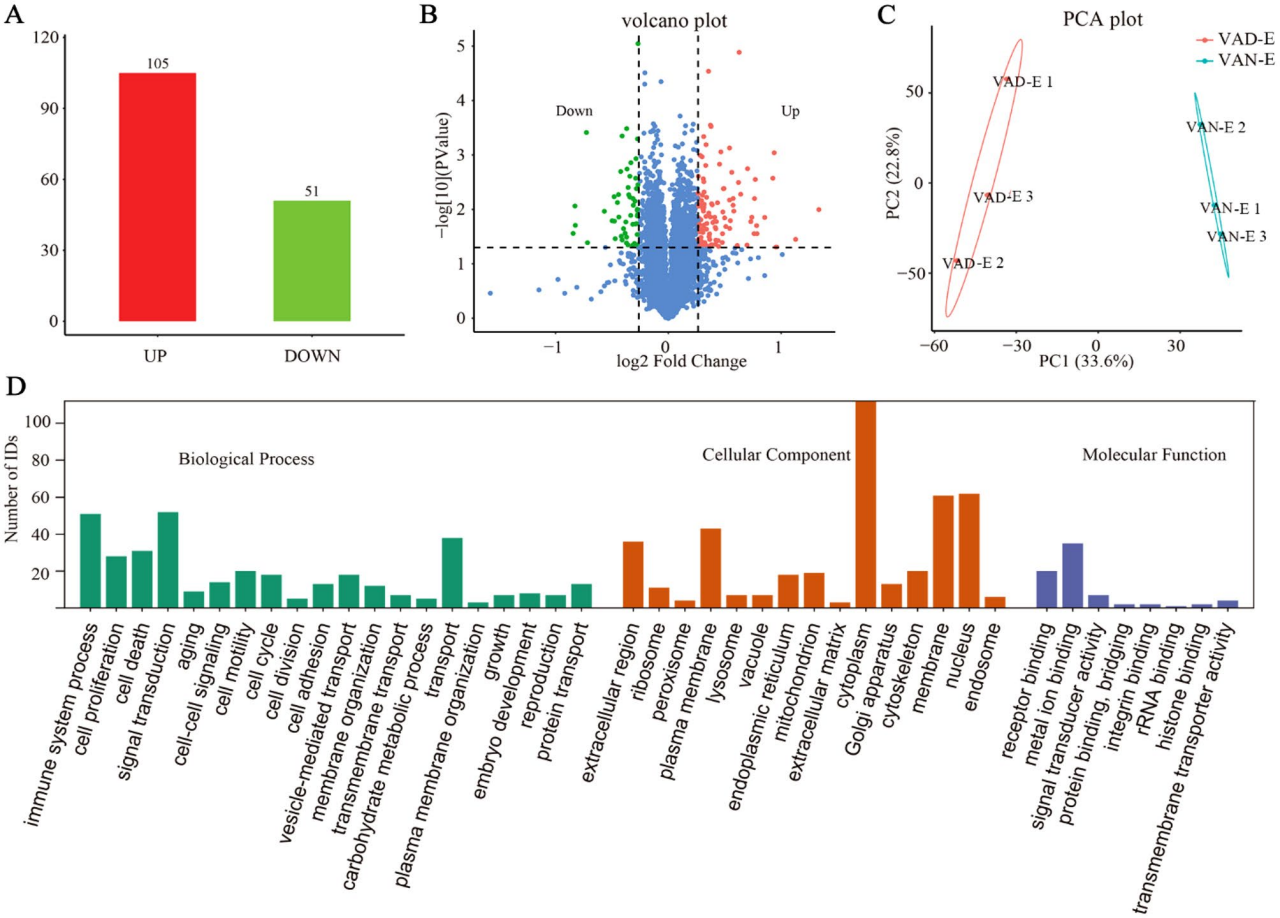
Differential proteins between the VAN-E and VAD-E groups. (**A**) Statistical analysis of the number of differential proteins in samples. (**B**) Statistical analysis of the number of differential proteins in samples. (**C**) PCA. (**D**) GO analysis. n = 3.

**Figure 7:**
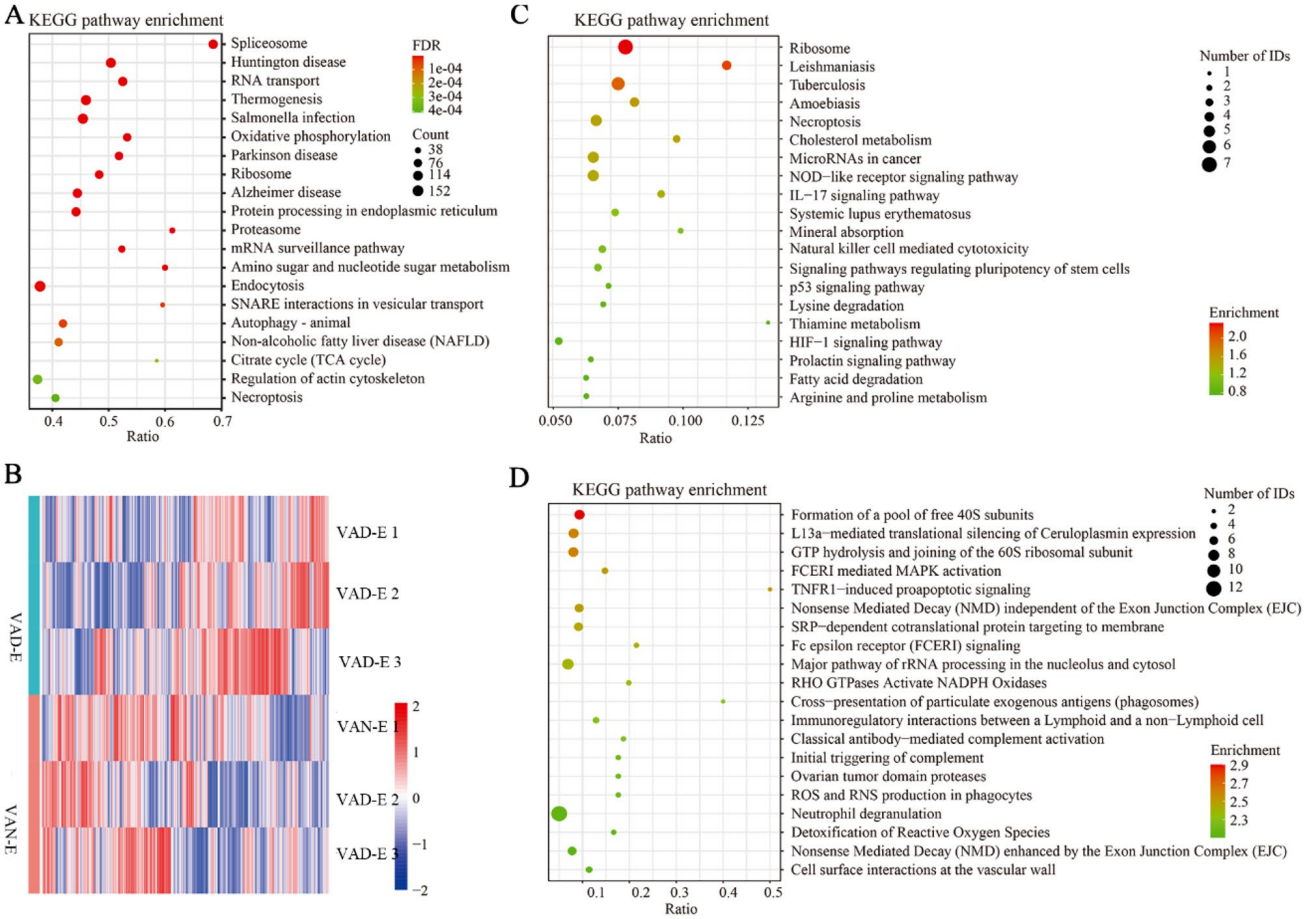
Differential protein enrichment analysis (**A**) KEGG pathway enrichment. (**B**) KEGG pathway enrichment. (**C**) Global analysis clustering heatmap. (**D**) Reactome pathway enrichment.

Next, we conducted proteomics analysis to comprehensively evaluate differential protein expression between the VAN-E and VAD-E groups. The processed data are showed in the supplementary table 2. In short, proteins with a significant quantitative change (P < 0.05) and a fold-change of 3 were regarded as significantly differentially expressed proteins (DEPs) between the two groups. A total of 156 proteins varied in abundance and were related to the quantified peptides, among which 105 proteins exhibited increased levels and 51 proteins exhibited decreased levels (Fig. 6A). Volcano plots and the PCA results showed large variations in protein abundance between the VAD-E and VAN-E groups (Fig. 6B,C). Moreover, the DEPs were related to cellular components, including extracellular space and extracellular region, while they were related to antigen binding antioxidant activity and peptidoglycan immune receptor activity in molecular function (Fig. 6D). The KEGG pathway enrichment analysis showed that the DEPs were related to functions involving ribosomes, leishmaniasis, the Fc epsilon RI signaling pathway, vitamin digestion and absorption, tuberculosis, amoebiasis, necroptosis, and cholesterol metabolism (Fig. 7A–D). The results showed that DEPs were mainly related to biological processes, including the innate immune response, response to bacteria, and response to aldosterone (Fig. 6D). Notably, the results indicated that the ribosome was the most statistically significant differential pathway. Our results showed that VAD could influence the function of ribosomes, which can be considered targets for VAD in ARDS. In addition, other pathway differences between the VAD-E and VAN-E groups were mainly related to metabolic pathways, inflammation and immunity, which may be of particular interest in research on the role of VAD in ARDS.

### Metabolome analysis

**Figure 8:**
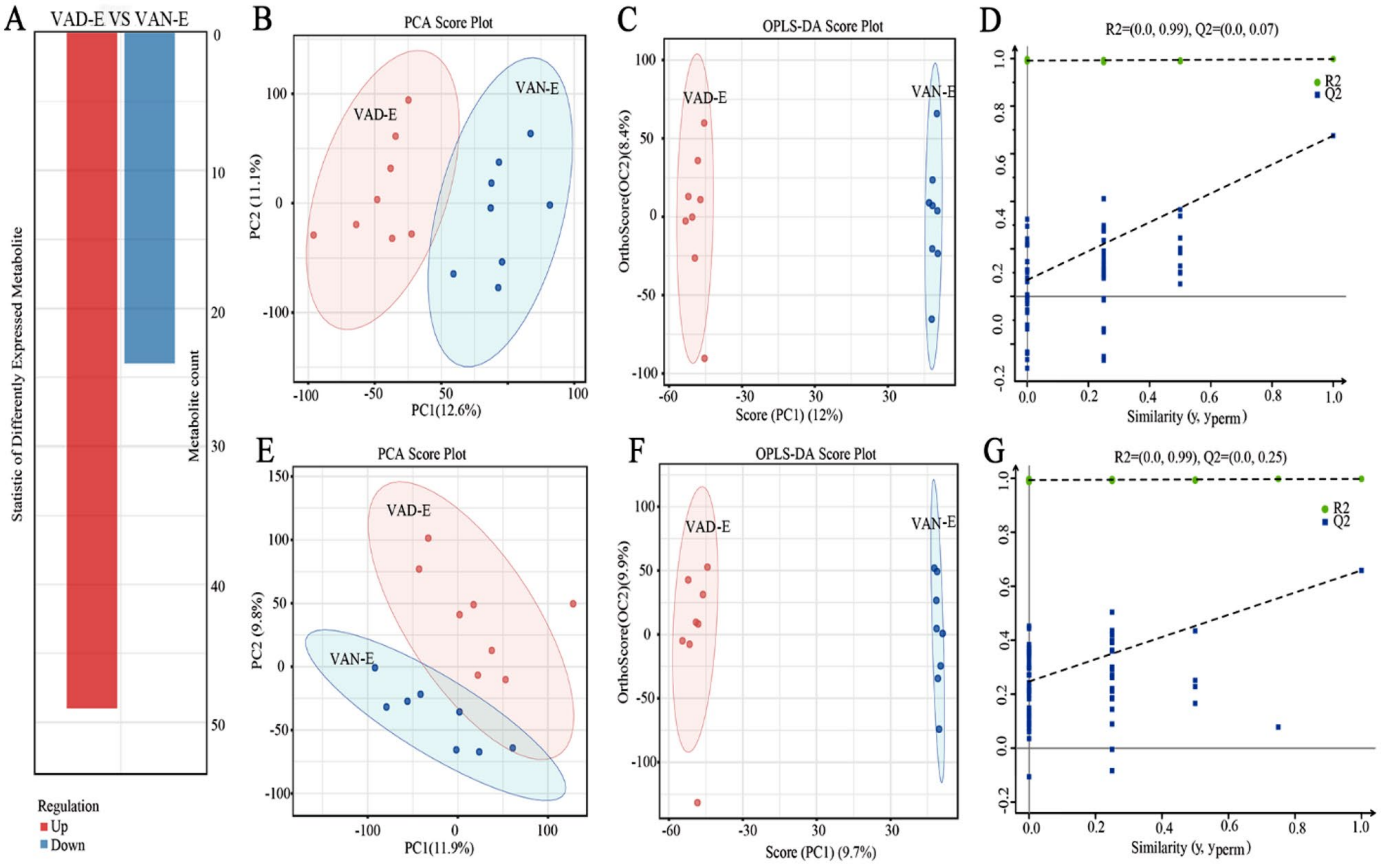
Characterization of the metabolome between the VAD-E and VAN-E groups. (**A**) Statistical histogram of differential metabolites between the two group. (**B**) PCA score plot of negative ions. (**C**) OPLS-DA score plots of negative ions. (**D**) PLS-DA score plot of negative ions. (**E**) PCA score plot of positive ions. (**F**) OPLS-DA score plots of positive ions. (**G**) PLS-DA score plot of positive ions. n = 8.

**Figure 9:**
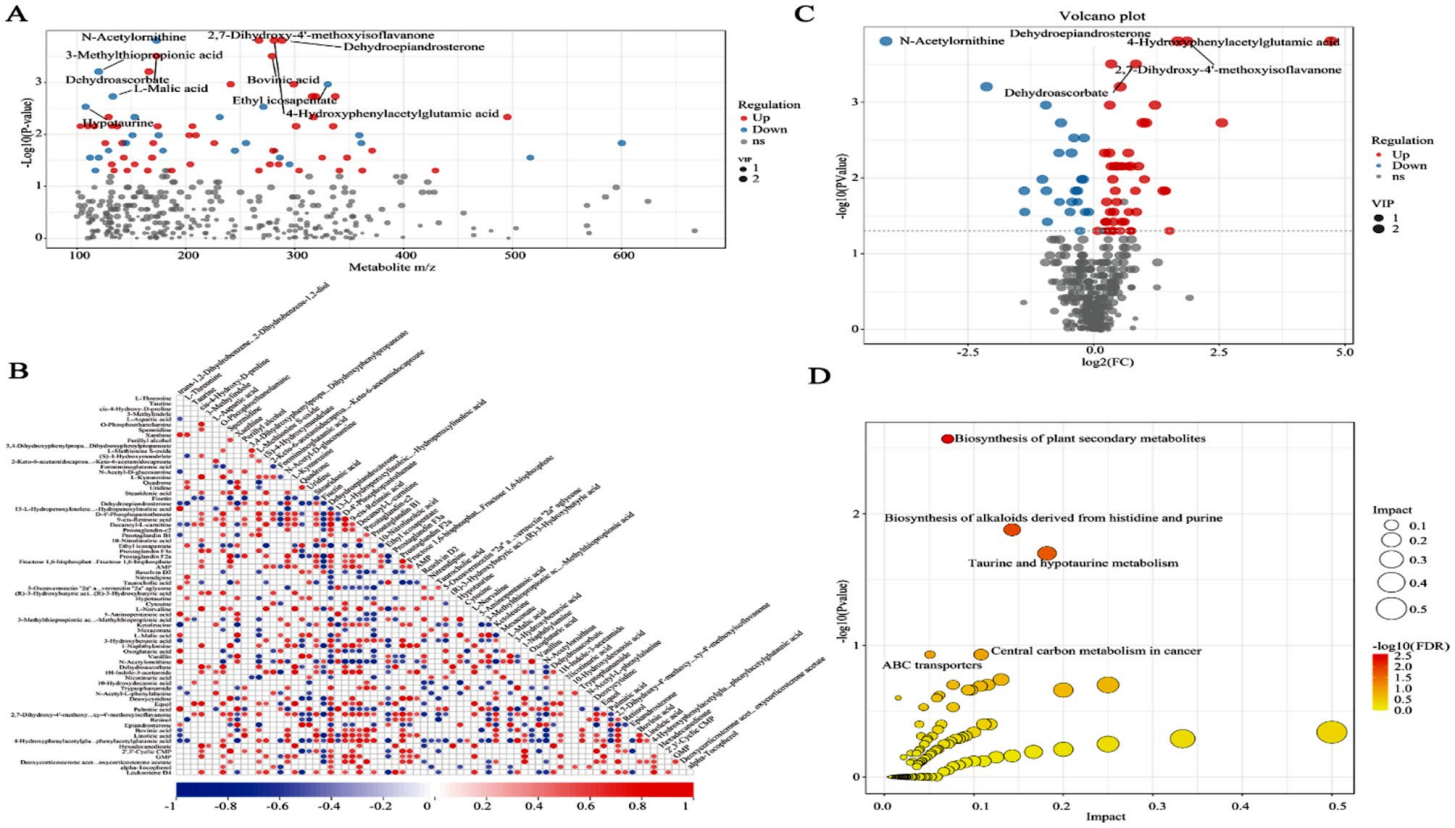
Metabolite enrichment analysis. (**A**) Scatter plot of the charge ratios and P values of differential metabolites. (**B**) Volcano plot of differential metabolites. (**C**) Differential metabolite association heatmap. (**D**) Bubble chart showing the metabolic pathways of influencing factors.

**Figure 10:**
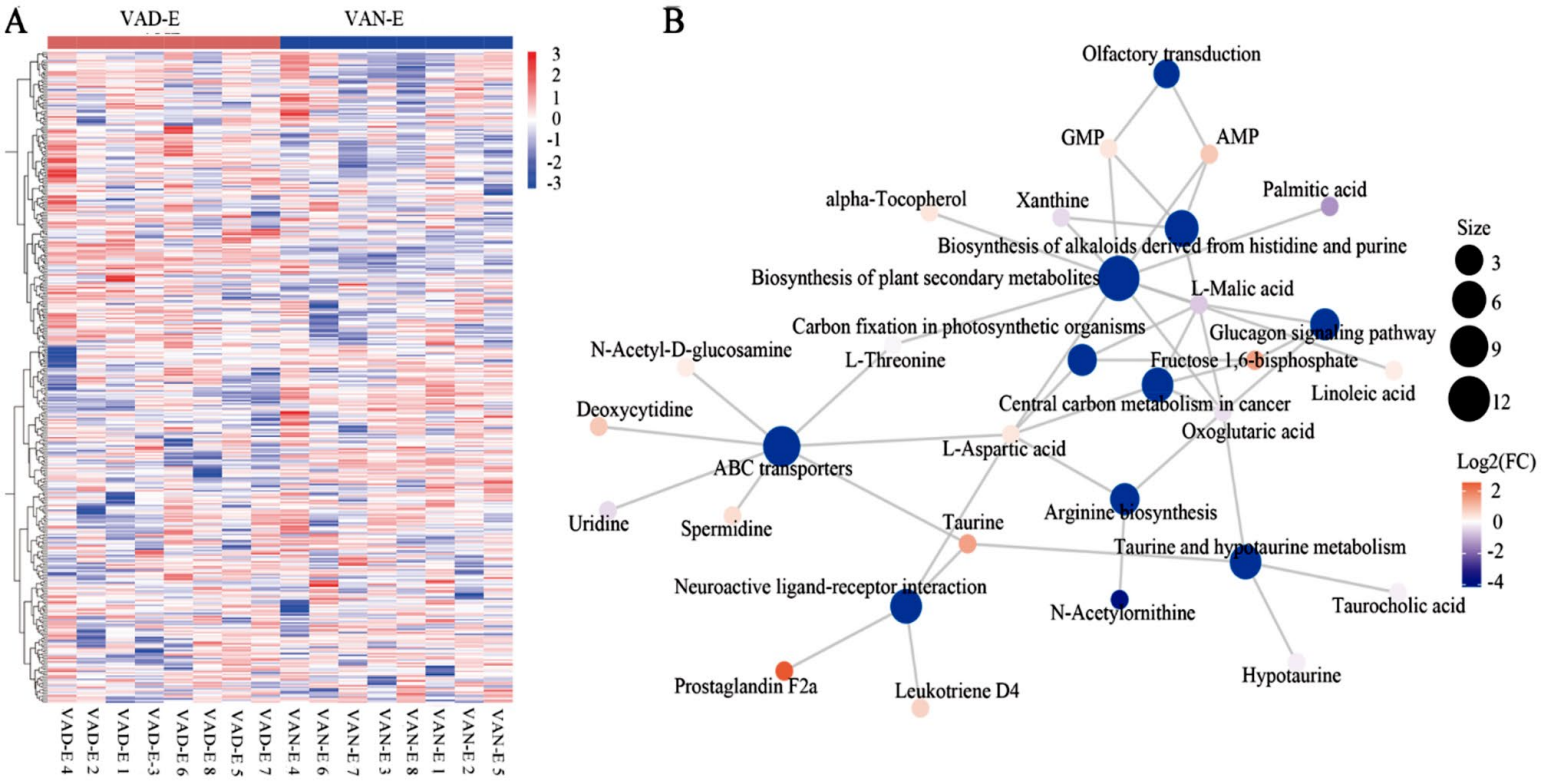
Differential metabolite analysis. (**A**) Hierarchical clustering heatmap of differential metabolites. (**B**) Z-score diagram.

**Figure 11:**
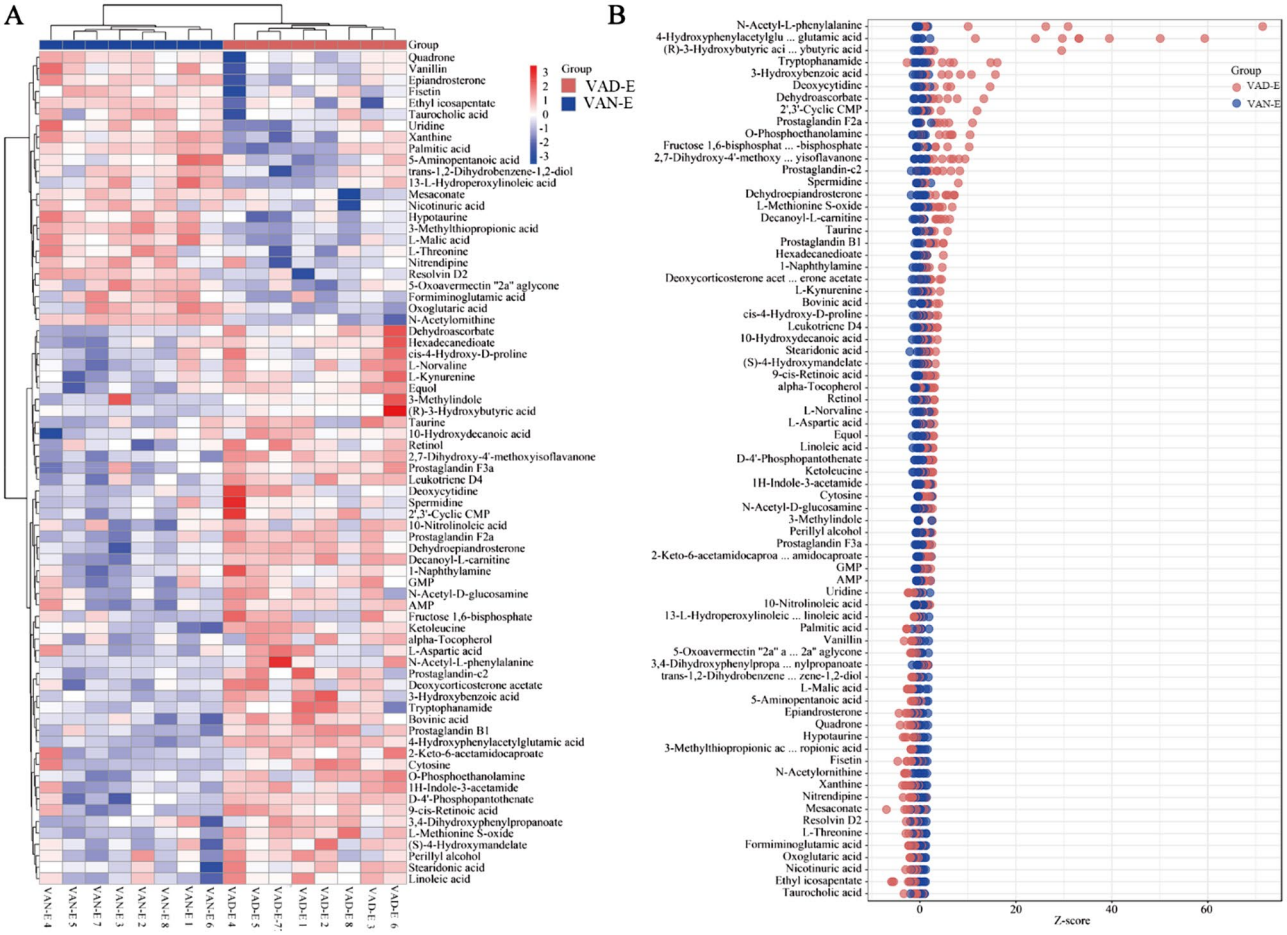
Metabolomic analysis. (**A**) Hierarchical clustering heatmap of differential metabolites. (**B**) Metabolic network diagram.

Metabolomic analysis was performed to assess the alterations in ARDS rats after VAD experiment. The processed data are showed in the supplementary table 3. Overall, 73 metabolites were differentially expressed, among which 24 metabolites exhibited decreased levels while 49 metabolites exhibited increased levels (Fig. 8A). Metabolite levels in the ARDS rat model subjected to VAD were different from those in the VAN-E group (Fig. 8B–G). The major differential metabolites were trans-1,2-dihydroben zene-1,2-diol, L-threonine, taurine, cis-4-hydroxy-D-proline, and 3-methylindole (Fig. 9A,B). The KEGG pathway enrichment analysis results indicated that the altered metabolites were mainly involved in the biosynthesis of plant secondary metabolites, biosynthesis of alkaloids derived from histidine and purine, the Fc epsilon RI signaling pathway, vitamin digestion and absorption, the HIF-1 signaling pathway, taurine and hypotaurine metabolism (Fig. 9C, D). The network diagram shows the interactions among the metabolite profiles (Figs. 10, 11). In short, the enriched pathways affected by VAD experiment were determined by comparison with those in the VAN-E group, and these pathways could be regarded as candidates for elucidating the effect of VAD on the mechanism underlying ARDS.

### Integrated multiomics analysis

**Figure 12:**
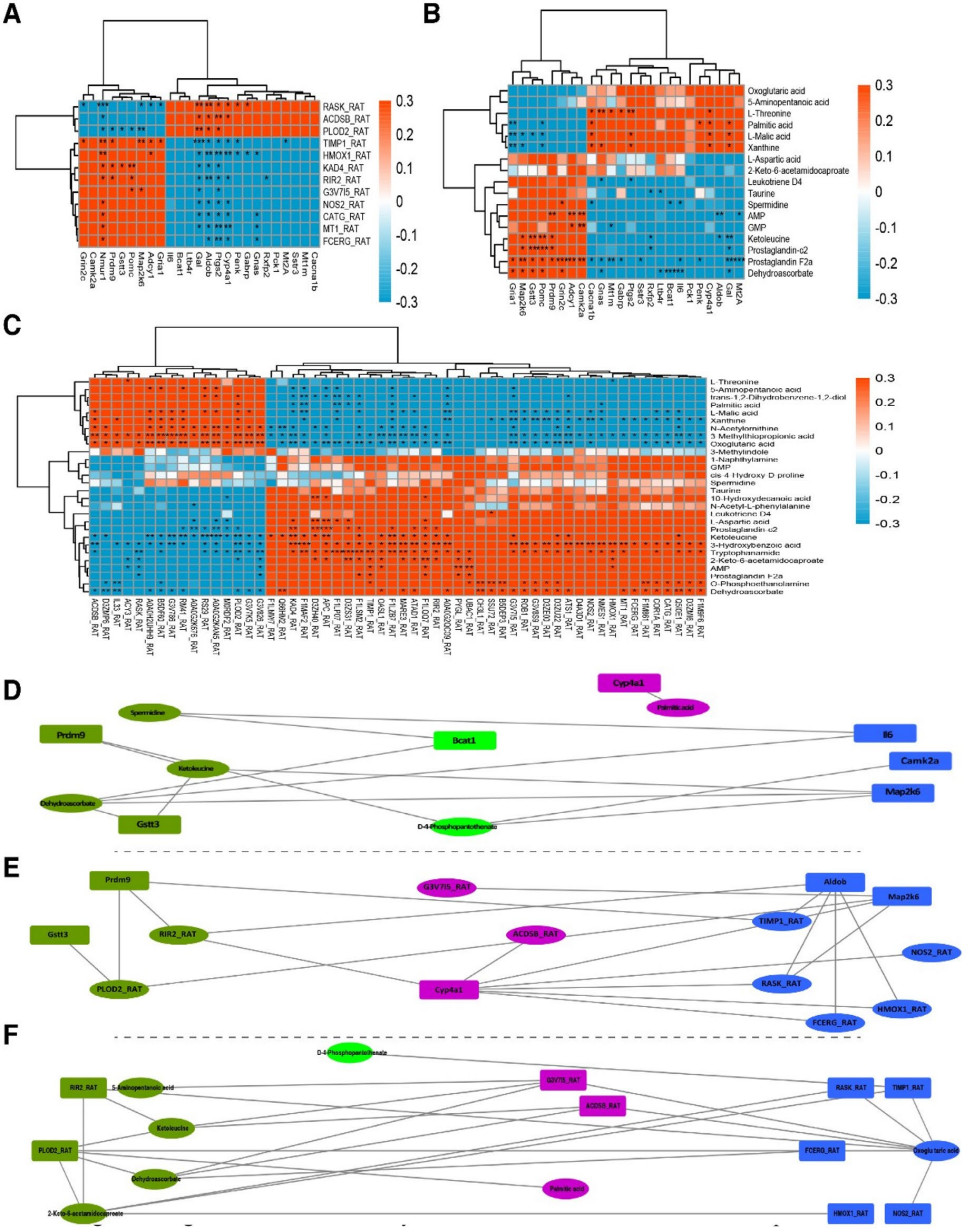
Integrated multiomics analysis of the effect in ARDS. (**A**) Transcriptional- and protein-associated heat map. (**B**) Thermography of transcriptomics and metabolomics. (**C**) Proteomics- and metabolomics-related heat map. (**D**) Association network diagram of transcriptomics and proteomics. (**E**) Association network diagram of transcriptomics and metabolomics. (**F**) Proteomics and metabolomics correlation network diagram.

In short, many genes, proteins, and metabolites were identified, and the results deserve further attention and in-depth functional study to elucidate the effect of VAD on ARDS. Combined multiomics analysis can be used to simultaneously explore biological problems from the two levels of "cause" and "effect"; verify the results of each assay; identify key genes, proteins, metabolites and metabolic pathways from a large amount of data; and further carry out follow-up research and application to fully elucidate an understanding of the regulatory mechanism of biological systems^19^. Our data indicated a high degree of posttranscriptional regulation in the VAD ARDS rat model. The KEGG analysis showed that the overlapped genes and proteins are mainly involved in the top relevant pathways, including the JAK2_RAT, RASK_RAT, HMOX1_RAT, and Fc epsilon RI signaling pathways, vitamin digestion and absorption, and the HIF-1 signaling pathway (Fig. 12A). In addition, the transcriptomics and metabolomics integrated analysis indicated the involvement of arachidonic acid metabolism, the Fc epsilon RI signaling pathway, vitamin digestion and absorption, the HIF-1 signaling pathway, and alanine, aspartate and glutamate metabolism(Fig. 12B). Moreover, the proteomics and metabolomics integrated analysis indicated that the overlapped KEGG pathways are significantly associated with arginine and proline metabolism, the Fc epsilon RI signaling pathway, vitamin digestion and absorption, the HIF-1 signaling pathway, pyrimidine metabolism, histidine metabolism, ascorbate and aldarate metabolism (Fig. 12C). Some important signal transduction pathways were also involved. Taken together, we integrated transcriptomics, proteomics, and metabolomics for comprehensive analysis and found that the coenriched pathways included the Fc epsilon RI signaling pathway, HIF-1 signaling pathway, glutathione metabolism, and alanine, aspartate and glutamate metabolism (Fig. 12D–F). In the Fc epsilon RI signaling pathway, the rask and fcerc genes separately regulate the rask and fcerc proteins, which affect the regulation of leukotriene D4. Moreover, the hmox-1, nos2 and timp1 genes regulate the expression of their proteins and participate in the HIF-1 signaling pathway through the metabolism of exogenous acids. In addition, for glutathione metabolism, rir2-encoded protein may have participated in VAD-induced ARDS by regulating the metabolism of spermidine and dehydroascorbate. Finally, the g3v7i5 and acdsb genes participate in VAD-induced ARDS by regulating ketoleucine metabolism, which is mediated by the participation of their proteins in alanine, aspartate and glutamate metabolism pathways (Table 1). In short, the integrated multiomics analysis identified multiple genes and pathways that might be related to VAD in ARDS, which mainly include the Fc epsilon RI signaling pathway, HIF-1 signaling pathway, glutathione metabolism, and alanine, aspartate and glutamate metabolism (Table 1). The identified genes, proteins and metabolites need to be further discussed and may play an important role in further applications.

**Table 1:** The integrative multiomic data analysis revealed genes, proteins and metabolites between the VAD-E and VAN groups with ARDS. The KEGG ID were cited from www.kegg.jp/kegg/kegg1.html.

KEGG ID	Pathway description	Genes	Pros	Metabolites
rno04664	Fc epsilon RI signaling pathway	RASK;FCERG	FCERG;RASK;	Leukotriene D4
rno04066	HIF-1 signaling pathway	HMOX1;NOS2;TIMP1	HMOX1;TIMP1;NOS2	Oxoglutaric acid
rno00480	Glutathione metabolism	RIR2	RIR2	Spermidine;Dehydroascorbate
rno00280	Valine, leucine and isoleucine degradation	G3V7I5;ACDSB	G3V7I5;ACDSB	Ketoleucine

### Ethical statement

All experiments were performed in accordance with relevant guidelines and regulations. Besides, the study is reported based on the ARRIVE guidelines.

## Discussion

ARDS is one of the common critical clinical illnesses in neonates, and early prevention is important for improving the prognosis of neonatal ARDS^20^. VA is an essential nutrient involved in maintaining human metabolism. Perinatal VAD can not only affect the development of fetal immune organs but also increase the risk of lung disease infection^21^. Some studies have supported that patients develop ARDS with VAD in COVID-19^22^.

However, the critical question here is; Does ARDS develop due to VAD in these systemic diseases? Or does ARDS develop because these diseases cause VAD? So we build the VAD neonatal rats model to explore the potential molecular mechanism of VAD on ARDS. Restori et al found that the incidence of respiratory tract infection in children with VAD is higher than that in children with normal VA levels, the level of VA in children with repeated infection is reduced, and VA supplementation can improve the lung injury induced by neonatal Streptococcus pneumoniae infection^23^. In this article, our results showed that the serum VA content in the VAD-E group was significantly lower than that in the VAN-E group, and the general condition of the VAD rats was poor. These obtained datas were consistent with the existing research results, suggesting that VAD could affect neonatal growth, development and immune function. In this study, we found that the VAD-E group showed more severe lung damage, tissue edema, necrosis, accumulation of inflammatory cells and immune cells than the VAN-E group, suggesting that VAD influences children, especially newborns. The severity of ARDS in children is closely related to VAD.

ARDS develops by triggering mechanisms that cause endothelial dysfunction as a result of VAD. Endothelial dysfunction and increased endothelial permeability, which are the fundamental disorders underlying ARDS.Moreover, LPS may cause deterioration in retinoic acid activity and surfactant synthesis and the development of ARDS through effecting type-II alveolar cells. In this study, we use LPS to build neonatal VAD ARDS models. LPS causes inflammatory cytokine discharge through NF-kB activation through the TLR4 and MyD88 pathways, thus causing organ damage and ARDS, which was described by Sarohan as a pathway in which VAD in the context of retinoic acid depletion syndrome in ARDS^22^.

RNA-seq results showed that the differentially expressed genes were involved in cytokine‒cytokine receptor interactions, protein digestion and absorption, the cAMP signaling pathway, Fc epsilon RI signaling pathway, HIF-1 signaling pathway, glutathione metabolism, alanine, aspartate and glutamate metabolism. The above signaling pathways can regulate certain cellular functions in physiological conditions, including cell proliferation, survival, and apoptosis. Overall, VAD experiment aggravated inflammation and immune system damage in ARDS. The transcriptome results may be regarded as a starting point for further mechanistic analysis and potentially represent candidates for biomarker exploration.

The proteome indicated 73 proteins that were significantly affected by VAD experiment in ARDS rats. The results showed that the DEPs were mainly involved in biological processes including the innate immune response, response to bacteria, and response to aldosterone, the Fc epsilon RI signaling pathway, HIF-1 signaling pathway, glutathione metabolism, alanine, aspartate and glutamate metabolism, which indicates that VAD treatment could aggravate ARDS through the innate immune response. The metabolites identified in VAD rats are mainly associated with the biosynthesis of plant secondary metabolites, the Fc epsilon RI signaling pathway, HIF-1 signaling pathway, glutathione metabolism, and alanine, aspartate and glutamate metabolism, which are mainly enriched in inflammation, immunity and metabolism.

Multiomics technologies are considered useful tools for molecular profiling and the clarification of complex systems biology^20^.Our multiomics results indicated that the enriched genes, proteins and metabolites are mainly involved in the Fc epsilon RI signaling pathway, the HIF-1 signaling pathway, glutathione metabolism, and valine, leucine and isoleucine degradation.

In short, HIF-1 signaling pathway, another signaling pathway discovered by researchers in relation to VAD, is closely related to ARDS conditions that previously occurred during some other acute clinical conditions such as preeclampsia, characterized by increased endothelial permeability. It is known that the HIF-1 signaling pathway, which increases as a result of hypoxia, has a protective effect on vascular stability and endothelial permeability through the increase in VEGF^24^. Likewise, it is known that VEGF gene expression is regulated by the genomic activity of retinoic acids. Therefore, we predict that endothelial stability and capillary permeability will deteriorate as a result of VAD, with disruption of VEGF synthesis and activity. Regardless of the etiological reason, the basis of ARDS is endothelial dysfunction and increased capillary permeability. With the increase in capillary permeability, intravascular proteins and fluids extravasate, causing edema and loss of function in the tissues. Besides, retinol depletion as the basic mechanism underlying the endothelial dysfunction and increase in endothelial permeability underlying ARDS. In this regard, we consider the HIF-1 signaling pathway, which was revealed in relation to VAD through VAD-VEGF-ARDS.

Besides, the Fc epsilon RI signaling pathway discovered by researchers is associated with anaphylactic type ARDS and respiratory distress, known as Type-I hypersensitivity reaction, which involves immunoglobulin E (IgE)-mediated release of antibodies against soluble antigens and inflammatory cytokines^25^. This reaction causes mast cell degranulation and the release of histamine and other inflammatory mediators, which leads to ARDS situation occurring in type-I anaphylactic reaction. Furthermore, glutathione metabolism contributes to the induction of trained immunity and may affect the inflammation threshold^26^. Moreover, research has found that reducing the contents of leucine, isoleucine and valine in foods can improve metabolism, regulate fat and sugar metabolism, and then regulate diabetes and obesity^27^. Khanfar et.al found that glutathione depletion may have a central role in COVID-19 mortality and ARDS pathophysiology. Therefore, elevating the GSH level in tissues may decrease the severity and mortality rates of COVID-19 related ARDS^28^. Grunwell et al found valine, leucine, and isoleucine biosynthesis can be identified as a metabolic signature in children with pediatric acute hypoxemic respiratory failure^29^. VAD can reduce fat and sugar metabolism through valine, leucine and isoleucine degradation pathways, affecting the weight, growth, immunity and development of neonates with ARDS. What’s more, research also showed that Ethyl ferulate ameliorates LPS-induced ARDS in an AMPK/Nrf2/NOS2-dependent manner^30^. Besides, HMOX1 variation may modulate ARDS risk through the promoter microsatellite polymorphism in ARDS^31^. In short, VAD may affect carbohydrate and fat metabolism through valine, leucine and isoleucine degradation signaling pathways, reducing the weight of newborn rats and affecting their growth and development. Moreover, VAD could affect the body inflammation, endothelial dysfunction and immune thresholds through the Fc epsilon RI and HIF-1 signaling pathways, regulating oxidative stress through glutathione metabolism and thereby aggravating ARDS lung injury. Moreover, our results integrated -omics data for the systematic description of biological systems, which may provide a useful approach to develop new strategies for the prevention, rapid diagnosis, and molecular mechanisms of VAD in ARDS.

### Supplementary Information

Supplementary Information 1.

Supplementary Information 2.

Supplementary Information 3.

Supplementary Information 4.

## Data Availability

All data generated or analysed during this study are included in this published article and its supplementary information files.
